# Roadside Exposure and Inflammation Biomarkers among a Cohort of Traffic Police in Kathmandu, Nepal

**DOI:** 10.3390/ijerph16030377

**Published:** 2019-01-29

**Authors:** Kabindra M. Shakya, Richard E. Peltier, Yimin Zhang, Basu D. Pandey

**Affiliations:** 1 Villanova University, Department of Geography and the Environment, Villanova, PA 19085, USA; 2 University of Massachusetts, Department of Environmental Health Science, Amherst, MA 01003, USA; rpeltier@schoolph.umass.edu; 3 Villanova University, Department of Mathematics and Statistics, Villanova, PA 19085, USA; yimin.z@villanova.edu; 4 Kathmandu and Everest International Clinic and Research Center, Sukraraj Tropical and Infectious Disease Hospital, Kathmandu 9045, Nepal; drbasupandey@gmail.com

**Keywords:** roadside exposure, air pollution, inflammation biomarker, Nepal, PM_2.5_

## Abstract

Air pollution is a major environmental problem in the Kathmandu Valley. Specifically, roadside and traffic-related air pollution exposure levels were found at very high levels exceeding Nepal air quality standards for daily PM_2.5_. In an exposure study involving traffic police officers, we collected 78 blood samples in a highly polluted spring season (16 February 2014–4 April 2014) and 63 blood samples in the less polluted summer season (20 July 2014–22 August 2014). Fourteen biomarkers, i.e., C-reactive protein (CRP), serum amyloid A (SAA), intracellular adhesion molecule (ICAM-1), vascular cell adhesion molecule (VCAM-1), interferon gamma (IFN-γ), interleukins (IL1-β, IL-2, IL-4, IL-6, IL-8, IL-10, IL-12, IL-13), and tumor necrosis factor (TNF-α) were analyzed in collected blood samples using proinflammatory panel 1 kits and vascular injury panel 2 kits. All the inflammatory biomarker levels were higher in the summer season than in the spring season, while particulate levels were higher in the spring season than in the summer season. We did not find significant association between 24-hour average PM_2.5_ or black carbon (BC) exposure levels with most of analyzed biomarkers for the traffic volunteers working and residing near busy roads in Kathmandu, Nepal, during 2014. Inflammation and vascular injury marker concentrations were generally higher in females, suggesting the important role of gender in inflammation biomarkers. Because of the small sample size of female subjects, further investigation with a larger sample size is required to confirm the role of gender in inflammation biomarkers.

## 1. Introduction

Air pollution is an important environmental health challenge across the world. Many studies have reported adverse health effects associated with roadside traffic exposures [1,2,3]. Air pollution has been linked with various health effects such as emergency room visits, childhood obesity [4], reduced lung function [3] preterm birth [5], autism [6], kidney disease [7], dementia [8], cardiovascular and respiratory illnesses, and overall mortality [9]. Studies have identified a large number of illnesses that are associated with air pollution exposure [10]. 

Exposure to air pollutants, such as fine particles, causes pulmonary inflammation and results in illnesses such as atherothrombosis [11,12,13]. Cytokines, a group of peptides and proteins, are related to inflammatory response to particulate exposure [14]. Several inflammatory biomarkers have been found to be associated with particulate matter (PM) exposure [15,16,17]. Cytokines such as interleukin-6 (IL-6) have been shown to be significantly correlated with traffic-related air pollution [18]. Traffic-related exposure increases the risk of cardiovascular disease and populations exposed to traffic pollutants are linked to elevated inflammation biomarkers and blood pressure [19]. IL-8, IL-1β, and C-reactive protein (CRP) are found to increase after ozone exposure [20]. Occupational or environmental PM exposure increases the levels of intracellular adhesion molecule (ICAM-1) and CRP [21]. Occupational exposure among people working as taxi drivers are related with inflammation biomarkers [22]. A study among trucking industry workers has found positive association of sICAM-1 but no significant association of IL-6 and hs-CRP with occupational particulate exposure [23]. Most animal models to date have shown that increasing doses of PM_2.5_ enhance inflammation biomarker concentrations [24].

However, inconsistent results on specific biomarkers related to PM exposure have also been observed [25,26,27,28,29,30,31] and no statistical difference has been observed in cytokines levels after laboratory exposure to concentrated ambient particles (CAPs) [32,33]. There was no significant association between CRP, IL-6, and tumor necrosis factor (TNF-α) and CAPs exposure in a study in UK, US, and Canada [32,33,34] while a study in the Netherlands reported the positive association between PM_2.5_ or PM_10_ with CRP [35]. Enhanced levels of inflammatory markers, such as CRP, IL-6, and TNF-α, suggest the future risk of cardiovascular diseases [25]. Individual responses to exposure may also vary and may be related with genetic predisposition [36] and other factors. Many studies are done in a laboratory in a controlled setting using animals or cell assays. Other ambient exposure studies are reported from mainly developed countries. In a review of published studies of systemic inflammation markers in humans, Møller et al. [26] reported a total of 25 studies from Europe, 23 studies from North America and 11 studies from Asia. Studies from Asia were mainly from developed or rapidly developing nations (e.g., China, Singapore, Taiwan, Iran, India, and Israel). Among rural Indian women, higher levels of IL-6, IL-8, and TNF-α in sputum were found from the households using biomass fuel than the ones using a cleaner fuel, i.e., liquefied petroleum gas [37]. There are overall fewer studies in understanding inflammatory responses for population exposed to traffic-related pollutants [30]. This study aims to assess the biomarker levels among the population that are routinely exposed to high particle levels in a developing country. 

Kathmandu is the capital city of Nepal, and is undergoing rapid development. This has led to quickly increasing population, vehicles, and urbanization, thus leading to increased emission of air pollutants in the Kathmandu Valley. Because the major air pollution source in the valley is traffic, human exposures near busy roads are expected to be higher than those in other locations in the valley [38] and traffic police are likely to be high-risk groups for adverse effects [29]. To investigate the roadside exposure of air pollution and related health effects, a major field campaign was conducted in 2014. Roadside and residential particulate pollution, anthropogenic gases, and respiratory health effects associated with roadside exposure from the same study have been published elsewhere [3,39,40,41]. In summary, roadside exposures to PM_2.5_ and black carbon (BC) were related to reduction of lung function among traffic police. Though PM_2.5_ concentrations were greatly reduced during summer compared to those in spring, components such as BC and several elements were not much lower during summer compared to those in spring, indicating the important contribution of vehicular emissions in both seasons. Several studies [42,43] have reported the high level of particulate levels in the Kathmandu Valley. High particulate levels have also been reported from the other valley in Nepal [44]. The main objective of the current work is to assess the seasonal changes in inflammation biomarkers among traffic workers and analyze the association of biomarker concentrations with air pollution exposure. To our knowledge, this is the first study from Nepal analyzing a comprehensive suite of inflammation biomarkers to assess the effect of air pollution exposure. This study provides baseline data to compare the biomarker levels among the population at different environmental conditions in future studies.

Populations who work on roads such as taxi drivers are routinely exposed to high air pollution levels and are considered as high-risk groups [22]. Traffic police can also represent other roadside occupational exposures [45]. Higher chromosomal aberration frequencies in lymphocytes in Turkey [46] and higher biomarkers of inflammation/infection in Pakistan [47] are found in traffic police compared to those in control population. Monitoring biomarkers in such cohorts helps to investigate the effects of occupational exposure to pollution. Traffic police in the Kathmandu Valley work on roads and direct the flow of traffic because the studied area had no functioning traffic signals. These workers spend several hours per day in traffic and such exposures have been found to decrease the lung function after the occupational exposure [3]. Therefore, traffic police officers were selected to evaluate the occupational exposure of particulate pollution and the biomarker levels. We hypothesized that particulate levels will be associated with levels of inflammation biomarkers among traffic police officers in the Kathmandu Valley.

## 2. Method

### 2.1. Study Population

A total of 53 traffic volunteers were recruited for this study. Prior to the study, approval for the study was taken from the institutional review board at the University of Massachusetts, Amherst and Nepal Health Research Council, Nepal. Permission to carry out the study was also completed from the government of Nepal. A total of 36 traffic volunteers participated in air pollution exposure study in the spring season, and 30 traffic volunteers participated in the summer season, though not all subjects consented to provide blood samples, where 33 volunteers permitted blood samples in spring, and 29 volunteers permitted blood samples in summer. These samples were from 29 men and 4 women in spring, and 25 men and 4 women in summer. From the subjects who volunteered to provide blood samples, there were 13 smokers and 14 non-smokers in the spring season and there were 9 smokers and 21 non-smokers in the summer season. All the subjects were prescreened for their medical history of asthma, serious respiratory or heart diseases, tuberculosis, and diabetes, and these were used as exclusion criteria for participation in this study. Descriptive statistics of subject demographics are given in Table 1. 

### 2.2. Sampling

This study was conducted in two phases: spring (16 February to 4 April 2014) and summer (20 July to 22 August 2014). Six sites were selected for the study: Kalanki, Balaju, Chabahil, Koteswor, Thapathali, and Jawalakhel. These sites were selected because they were the busiest traffic intersections in the valley. Detailed descriptions of the sampling have previously been published [3,40]. Briefly, at each site, personal exposure of particulate pollution was monitored for six traffic volunteers for up to six days (Sunday to Friday). Sunday is a working day in Nepal. While working on the road, each of the six traffic volunteers carried a bag containing air pollution monitors. These six traffic volunteers worked around the vicinity of each of the six sites. We followed thirty-six traffic volunteers from six sites for six weeks during spring and thirty traffic volunteers from five sites during summer. Volunteers were requested to wear N-95 masks for half of the week (3 days) as an intervention component of the study, where personal exposure to ambient pollutants was dramatically decreased. At each site, all volunteers were requested to wear masks either on the first half of the week (Sunday to Tuesday) or on the second half of the week (Wednesday to Friday). This was done to avoid confounding effects between the use of mask and day of the week. In general, all volunteers have a similar number of working hours (8–10 h), with two working shifts: morning and afternoon. The average temperature during sampling periods in spring and summer was 14.8 °C and 23.6 °C, respectively; relative humidity in spring and summer was 73.2% and 88.0%, respectively; total precipitations in spring and summer were 50.47 and 266.6 mm, respectively [40].

### 2.3. Blood Sample Collection and Biomarker Analysis

Blood samples were collected by a professional phlebotomist working at a local hospital. Three blood samples per subject were taken in the beginning, middle, and end of the week separately. In total, there were 78 blood samples from 33 traffic volunteers in the spring season and 63 blood samples from 29 traffic volunteers in the summer season. Blood samples were centrifuged for 15 min within 24 h of sample collection. After centrifugation, serum samples were stored in a freezer at −20 °C. Standard deep freezers at –80 °C were not available, and are, in fact, rare in Nepal. Samples were then transported under refrigeration to our laboratory at the University of Massachusetts, and then stored at −80 °C. No sample quality checks were performed in this set of samples to assess sample degradation. 

V-PLEX assay kits (Mesoscale Discovery, Rockville, MD, USA) were used for biomarker analysis. Proinflammatory Panel 1 (human) kits were used for analyzing 10 cytokines: interferon gamma (IFN-γ), interleukins (IL-1β, IL-2, IL-4, IL-6, IL-8, IL-10, IL-12p70, and IL-13), and tumor necrosis factor (TNF-α) (Table 2). Vascular injury panel 2 (human) kits were used for analyzing serum amyloid A (SAA), CRP, vascular cell adhesion molecule (VCAM-1), and intercellular adhesion molecule (ICAM-1). Protocols from the respective kits were followed to analyze the biomarkers on Discovery Workbench (Mesoscale Discovery, Rockville, MD, USA).

### 2.4. Air Pollution Exposure

Real-time exposure levels of PM_2.5_ and BC were measured from the individual subjects for 5–6 days using devices that were carried by participants. A nephelometer (pDR-1500, Thermo Scientific Inc., MA, USA) and a microaethalometer (AE51, AethLabs, CA, USA) were used to measure PM_2.5_ and BC levels, respectively. Particulate samples were collected by polyflourotertaethylene (PFTE) filters for 24 h on nephelometers for post hoc analyses. The filters were analyzed for water-soluble ions by ion chromatography and for elements by X-ray fluorescence spectroscopy. Results for the air pollution and chemical components were published elsewhere [40]. Sampling lines used an inlet affixed to the breathing zone of a participant.

### 2.5. Data Analysis

**Figure 1:**
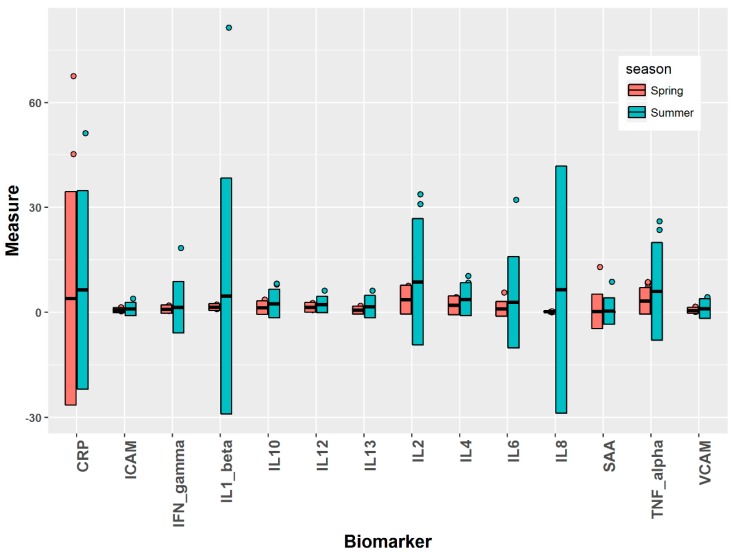
Concentrations of biomarkers during spring and summer seasons, 2014. CRP, SAA, ICAM-1, and VCAM-1 are shown in µg/mL; IFN-γ, IL1-β, IL-2, Il-4, IL-6, IL-8, IL-10, IL-12, IL-13, and TNF-α are given in pg/mL. In the figure, the central line across the box represents the mean, and the upper and lower boundaries correspond to three standard deviations above and below the mean, respectively. The outliers falling outside three standards of the mean are marked with circles. For ease of illustration, four biomarkers are rescaled: IL-4 upscaled by a factor of 10; SAA and IFN-γ downscaled by a factor of 1/20; IL-8 downscaled by a factor of 1/100.

Figure 1 shows the summary statistics of biomarker results in the spring and summer seasons of 2014. For each biomarker, outliers were identified by falling outside three standard deviations of the means for spring and summer separately. The means and standard deviations were calculated by assigning equal weights to each data entry (an individual could have multiple data entries if they consented to provide blood samples at multiple days of the week). These outliers were hence removed and the resultant data were used for all subsequent analysis. As a result, no data were removed for IL-1β and IL-12; <1% of data were removed for IFN-γ, IL-2, IL-4, IL-6, IL-13, TNF-α, and ICAM-1; <2% of data were removed for IL-8, and IL-10; 4% data were removed for VCAM-1; 5% of data were removed for SAA, and 6% of data were removed for CRP.

To further investigate variations in biomarker measurements, linear mixed regression models were built, with each type of biomarkers as the response variable and the following factors as potential explanatory variables, such as season, PM_2.5_, BC, gender, smoking, and the use of masks. The linear mixed model also made it happen to include the dependency structure of biomarkers from the same subjects who were followed in both seasons. Figure S1 (Supplementary Materials) illustrates the overall modelling process. 

First, a linear mixed model can be formulated as follows:

(1) $$y_{ij}=\beta_{0}+\beta_{1}x_{1}+\beta_{2}x_{2}+\ldots +\beta_{6}x_{6}+\eta_{i}+\epsilon_{ij},$$

(2) $$\eta_{i}\sim N\left(0,\;\sigma_{\eta }^{2}\right),\;\;\;\epsilon_{ij}\sim N\left(0,\;\mathrm{var}\left(\epsilon_{ij}\right)\right),$$

where $y_{ij}$ is the level of one biomarker from subject *i* at *j*th measurement and $x_{1},\ldots ,x_{6}$ are the explanatory variables, including PM_2.5_, BC, season (spring or summer), smoking (Y or N), gender (F or M) and mask (Y or N), with corresponding coefficients $\beta_{1},\ldots ,\beta_{6}$. The variables x_1_ to x_6_ are treated as fixed effects and $\eta_{i}$ denotes the random effect among subjects. Hence, the dependency between the biomarkers measured from the same subjects is included in the model by sharing the same term $\eta_{i}$. The term $\epsilon_{ij}\;$ is the random error in each measurement and its variance is assumed to be also associated with the explanatory variables, following the power-of-X dispersion function [48] specified below:

(3) $$\mathrm{var}\left(\epsilon_{ij}\right)=\;\sigma_{\varepsilon }^{2}\exp\left(\gamma_{0}+\gamma_{1}x_{1}+\gamma_{2}x_{2}+\ldots +\;\gamma_{6}x_{6}\right),$$

where $\sigma_{\varepsilon }$ is an unknown parameter and $\gamma_{k}\;\left(k=0,\;1,\;\ldots ,6\right)$ are unknown parameters called the dispersion effects parameters.

Second, model diagnostics were performed on the full model to investigate the need for transformation on the biomarker response variables and to assess outliers. Box-Cox transformations were applied on the biomarker variables, if necessary, to correct non-normality. To examine assumptions with the transformed model, scaled residuals, defined through the Cholesky decomposition of the variance-covariance matrix, were obtained in place of raw residuals, as they tended to be uncorrelated with the constant mean zero. To further detect outliers and potentially influential data points, restricted likelihood distance [49] was used as an overall influence measure in addition to scaled residuals. Specifically, a data point with either a restricted likelihood distance or a scaled residual more than three was identified as an outlier and removed from the dataset. Once an outlier was deleted, the model on the updated dataset was refitted and an outlier was removed one at a time until no more outliers were detected in the residual analysis.

Third, on the transformed full model, two stepdown variable selection procedures were employed partially based on *p*-values of the effects. Specifically, using the complete fixed effects under consideration, a stepdown selection on the dispersion effects only was performed, until the lowest Bayesian information criterion (BIC) [50] was achieved. Then keeping these selected dispersion effects, another stepdown selection on the main effects only was performed and BIC was used to select the final model. Note that the last model from the second stepdown selection did not necessarily have the lowest BIC. Thus, sometimes, some insignificant effects were kept in the final model to attain a lower BIC, or some significant effects might be excluded for the same reason. 

Finally, the adequacy of each final linear mixed model was checked by a residual analysis. As shown in Figure S1 (Supplementary Materials), outliers were identified and removed in the same manner until the model was free of outliers. The final results of the models for each biomarker could be found in Table 3.

## 3. Results

### 3.1. Air Pollution Exposure over Seasons

Table 1 compares particulate air pollution exposure between spring and summer. Independent two-sample t tests were conducted on PM_2.5_, BC and other measurements from passive sampling. From Table 1, PM_2.5_ concentrations were significantly lower in the summer season than in the spring season, a generally expected finding given the generally cleaner conditions in summer at this location. While the mean PM_2.5_ concentration was larger by a factor of 2.7 during spring compared to that in summer, the mean BC concentration was larger by a factor of 1.1 during spring compared to that in summer. Measurements were made from busy roads and roadsides, and the BC sources at these sites were related mainly with vehicular exhaust. Because traffic activities were not expected to be different during two seasons, the insignificant difference in BC concentrations suggested the importance of traffic-related PM sources in both seasons. The increased level of PM_2.5_ from summer to spring was due to the higher regional pollution in spring than in summer. There were also additional sources such as seasonal operation of brick kilns and refuse burning during the spring season. In contrast to particle pollution, nitrogen oxides, sulfur dioxide, and ozone measurements obtained from passive sampling were not significantly different between the two seasons. Although the PM_2.5_ level was shown to be associated with the season, they did not present highly overlapping power in explaining the biomarker response variables in the linear mixed models. It was shown that in the full model with the six independent variables, the variance inflation factors (VIFs) of PM_2.5_ and season, which measure the dependency among variables, were both below 4, where the multicollinearity issue usually arises when any of the VIFs exceeds 5 or 10. More model results about the effect of PM_2.5_ and season will be discussed in Section 3.3.

### 3.2. Biomarkers over Seasons

The comparison results of biomarker concentrations between spring and summer of 2014 are shown in Table 2. Three statistical tests were considered. The independent *t*-test performed a two-sample *t*-test assuming independence of the volunteer samples in the two seasons. However, there were repeated subjects who participated in the samples from both seasons. Unfortunately, we were not able to follow all subjects during both seasons. In fact, during the second phase of study in summer, more than half of subjects had been transferred to other locations. Only 13 subjects were repeated from the respective sites in both seasons. Out of these 13 repeated subjects, serum biomarker data were available for only 12 subjects. Hence, dependent samples only counted these 12 subjects. Then, a parametric two-sample *t*-test and a nonparametric Wilcox test were both performed on the dependent samples. All these three tests using independent or dependent samples were two-sided and conducted on the data of each individual’s average biomarker concentrations. From Table 2, out of 14 biomarkers, 10 of them showed significant increases, at the significance level of 0.05, in summer than in spring (consistent across all three tests), whereas VCAM-1 exhibited no significant differences in any of the three tests. Three biomarkers, CRP, ICAM-1, and IFN-γ, showed significant differences only partially from one or two of the tests. Of these ten biomarkers with consistently significant results, eight of them were very significant with *p*-values below 0.001 while assuming independent samples and five of them were very significant using dependent samples. The top three biomarkers with the overall most significant differences were IL-2, IL-4, and IL-6.

### 3.3. Effects of Air Pollution and Various Factors on Biomarkers

In the previous section, test results appeared to show that season had a strong impact on biomarker concentrations. However, those results did not take into account the potential effects of other factors, along with season, on biomarkers. Hence, linear mixed regression models were run to investigate the effect of six variables simultaneously on biomarker levels: PM_2.5_, BC, season, smoking habit, wearing mask, and gender. Table 3 lists the estimated coefficients of these variables as fixed effects in the linear mixed models for each biomarker. The results showed that PM_2.5_ exposure was positively associated with three biomarkers, i.e., CRP, SAA, and IL-10, and negatively associated with four biomarkers, i.e., IL-1β, IL-12, IL-13, and IFN-γ, while holding all the other factors in the models constant. BC did not have an effect on most of biomarkers except two biomarkers, where it was negatively correlated with two biomarkers, i.e., Il-10 and TNF-α, but the effect was very small (Table 3). As expected, season had a strong effect on biomarker levels and it was the only factor that showed a significant effect for all the biomarkers except IFN-γ. Consistently, the concentration levels across all these biomarkers tended to be higher in summer than in spring. 

**Figure 2:**
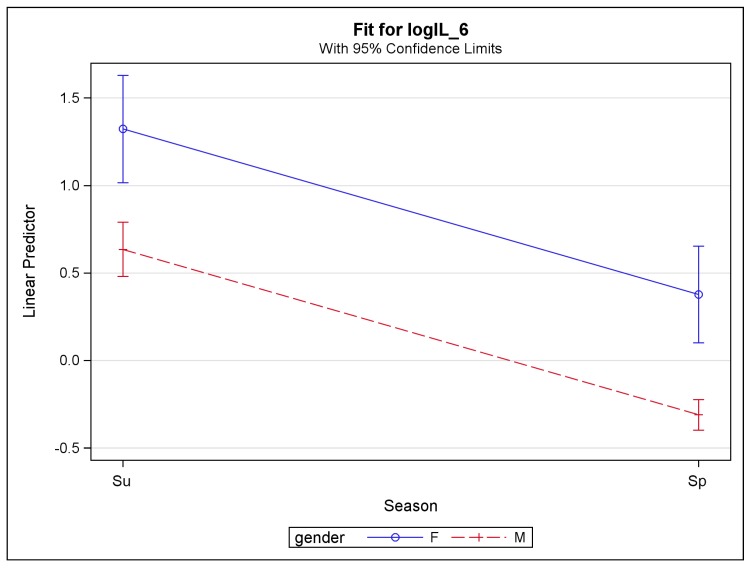
Predicted log (IL-6) concentration (with confidence intervals) versus season (Su: summer; Sp: spring) and gender (F: female; M: male).

Subject gender also had an effect on eight biomarkers: SAA, IL-2, IL-4, IL-6, IL-10, IL-12, IL-13, and IFN-γ. For these eight biomarkers, females tended to have higher concentration levels than males. Note that there were 7 female subjects out of 53 subjects. The effects of season and gender are illustrated in Figure 2 in the model for IL-6. In addition, it was found that wearing a protective facemask had a positive effect on IL-10, IL-12, and IL-13. The smoking habit was positively associated only with TNF-α with a *p*-value of 0.02.

**Figure 3:**
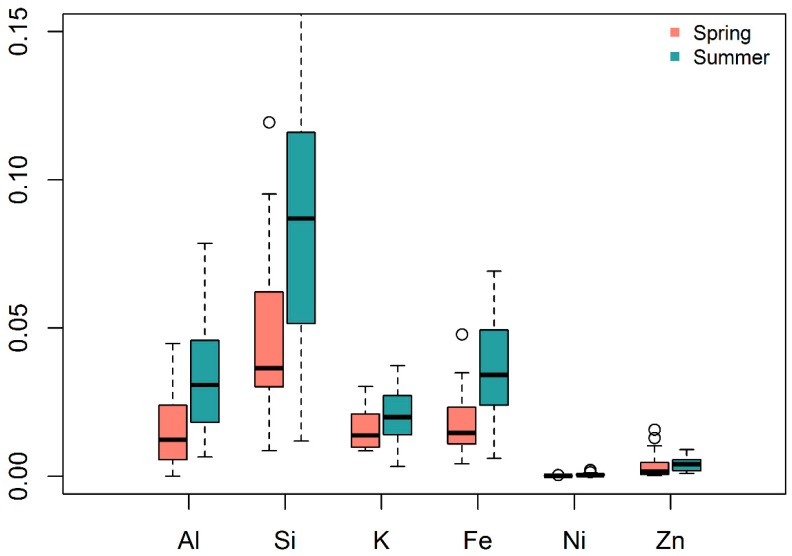
Normalized concentrations of elements (μg/m^3^ of elements normalized by μg/m^3^ of PM_2.5_) during spring and summer seasons, 2014.

Since the PM_2.5_ concentration did not exhibit a significant effect on more than half of the biomarkers from the linear mixed models, we considered to further investigate the PM_2.5_ chemical composition (elemental concentrations) and their relations with season on biomarkers. The results were plotted in Figure 3. It can be seen that the contributions of six elements—aluminum (Al), silica (Si), potassium (K), iron (Fe), nickel (Ni), and zinc (Zn)—were enhanced in summer than in spring. This showed that the variation among biomarker levels may be related to particulate composition more than the total particulate mass alone. Some of these elements such as Fe, Ni, and Zn are also related with traffic emissions [51], suggesting also the changes in traffic patterns in two seasons. However, there was no significant difference in BC concentrations between two seasons. Due to a large amount of missing values in element contributions, they were not included as explanatory variables in the linear mixed models of this study, but they could be valuable factors for biomarkers to be investigated in future studies. Besides these six elements, other elements were also measured on collected filters by X-ray fluorescence spectroscopy, but they were not enhanced during summer than during spring [40].

## 4. Discussion

### 4.1. Air Pollution Exposure

Exposure to PM_2.5_ was generally higher in the spring season than in summer in the Kathmandu Valley (Table 1), but the elemental species contribution of PM_2.5_ was found to be enhanced during summer than during spring (Figure 3). It was mainly PM_2.5_ that saw the reduction in summer. BC levels were not significantly different between two seasons, suggesting only minor differences in traffic-related PM sources during two seasons. BC, emitted from incomplete combustion of fossil fuels and biomass, is used as a tracer of combustion sources and has been used to investigate traffic pollution in urban areas [52,53,54]. All pollution measurements were taken from roadside at the busiest intersections, where traffic-related activities were the major source of PM_2.5_. There were also other sources such as dust resuspension, construction-related activities, refuse burning, brick-kiln, and these activities were minimized during summer than in spring. Meteorology might play an important role as well, and summer monsoonal conditions likely had an important determinative effect on PM_2.5_, a pollutant with a multitude of sources by decreasing concentrations through atmospheric washout, whereas BC was less impacted by washout. Temperature and total precipitation were lower in the spring season compared to in summer. Thus, a reduced atmospheric boundary layer height and reduced wind, lower temperature and low or no precipitation may have impacted the removal of PM_2.5_ during spring. Among the trace gases, ozone is slightly increased, but not significantly, during summer than in spring as it is formed through a photochemical process. 

### 4.2. Biomarkers

On average, biomarker levels were higher during summer than in spring, despite having lower PM_2.5_ levels. Statistical tests, after adjusting for factors such as smoker, age, and gender in the models, yielded mixed association between biomarkers and PM_2.5_. Some biomarkers (CRP, SAA, and IL-10) were positively associated while other biomarkers (IL-1β, IL-12, IL-13, and IFN-γ) were negatively associated with PM_2.5_. No significant association was found between PM_2.5_ and seven biomarkers (VCAM-1, ICAM-1, IL-2, IL-4, IL-6, IL-8, and TNF-α). Previous studies have reported mixed findings for the association of various biomarkers with PM_2.5_ exposures. A cross-sectional study among the healthy residents living in traffic congested areas in Thailand did not show significant association of PM_2.5_ with IL-8 [27]. In the Greater Boston Area, Alexeeff et al. [28] found significant association between BC and sICAM-1, but no significant association between BC and VCAM-1. Positive association between PM_2.5_ exposure and hs-CRP was found among traffic policemen in China [29] but negative association was found among workers at truck terminals in Northeastern US [30]. Lower levels of TNF-α were observed among adolescents living in a city with high PM_2.5_ levels than in less polluted city in Bulgaria, and they attributed this to inhibition of cytokine production by particulates [31]. Results from this study suggested that PM_2.5_ mass alone was not the sole important factor in affecting the biomarker levels on the studied subjects. There may be several other factors such as age, lifestyles, past environmental exposures, and ethnicity, which may have contributed to the variation of biomarkers. Among these factors, the following potential effects are discussed in subsequent paragraphs, such as (1) chemical composition, stress, and weather, (2) long-term occupational exposure, and (3) body fat and genetics.

Firstly, the higher levels of inflammatory biomarkers during the cleaner summer season might be due to relative fractions of PM chemical components rather than only PM mass concentration, which is consistent with other findings [14] that suggest that PM speciation might be more important than the PM concentration in determining biomarker changes in humans. For example, Carter et al. [55] attributed the increase in cytokines in human airway epithelia cells to the metals found in particles. Brucker et al. [56] found metals in blood were positively correlated with pro-inflammatory cytokines in taxi drivers. A similar effect may be playing a role in inducing inflammation and vascular injury markers in this cohort, even in the presence of the reduced bulk PM_2.5_ concentration. Alternatively, the pattern of biomarkers could also be attributed to other factors, for example season- and weather-related stress. Humidity and temperature was higher during summer than during spring. The sampling period in summer coincided with the monsoon season in Nepal. All the traffic volunteers were working on these busy roads to direct the traffic and prevent and control traffic jams at these busy intersections. There were no operating traffic lights at the sampling locations. We met two times a day (early morning before they go to work; late afternoon after they return from work) with each of traffic volunteers and we visibly observed higher stress during summer than during spring.

Secondly, it is also possible that inflammation biomarkers were already enhanced in these traffic volunteers because of their high occupational exposure and thus the inflammation markers that we measured were immune to short-term influence from PM_2.5_ personal exposure in our cohort study. Ying and Rajagopalan [57] suggest that the lack of association between short-term effects of PM exposure and inflammation biomarker does not necessarily mean that there is no effect from long-term exposure or there is no effect on other cytokine pathways. People working on roads such as taxi drivers have been observed with elevated levels of inflammation biomarkers compared to a control population [22].

Thirdly, it is also possible that the new recruits in summer may have other conditions causing high inflammation concentrations. Persons with excess body fat may have high inflammation biomarkers such as CRP and IL-6 [58,59]. However, mean BMIs were similar during two seasons (Table 1) and are not likely important here. Bind et al. [36] found genetics to play a role in chronic inflammation from air pollution exposure, though we lacked genetic information from this cohort. Certain population may have greater biological susceptibility or sociodemographic vulnerability [60]. Rückerl et al. [61,62] observed high association of inflammation biomarkers with air pollution in population with genetic susceptibility.

The study was conducted from six sites inside the Kathmandu Valley: Thapathali, Koteswor, Jawalakhel, Chabahil, Balaju, and Kalanki. The biomarker concentrations were not distributed evenly among the six sites studied. The highest biomarker concentrations were found in Thapathali, one of the cleaner locations in this study. Spatial variations among personal particulate exposure levels at six sites during two seasons are given in Table S1 (Supplementary Materials) and details are given in Shakya et al. [3,40]. As was shown from our model, gender had a significant effect on a large number of biomarkers (eight out of fourteen biomarkers), where female officers had higher concentrations of biomarkers compared to their male counterparts. Gender is known to play an important role in the degree of inflammation [63]. Other studies have shown higher biomarker levels in general in females than in males, e.g., CRP levels in the Dallas Heart Study [64]. Burnout, depression, and anxiety also affect differently inflammation biomarkers depending on gender [65]. Though female traffic volunteers were living together at a dormitory in the same house located at the sample sites, if they were still using biomass for cooking at their home, these dirtier indoor environment may contribute to higher inflammation biomarker levels [37]. That will also have more influence on females than males because females in Nepal are most likely to be responsible for cooking activities. Of all the six sites, Thapathali was the only site with all female traffic officers, all of whom were non-smokers, and consequently the highest biomarker concentrations were found in Thapathali. Because of allocation of females at only one site, we cannot discard the possibility of confounding effect of sites on gender. However, all the sites were located not very far from each other; distances among the six sites ranged from 3 to 11 km. The limitation of findings on gender is reiterated here due to very small female sample size. Further studies are needed before coming to conclusion regarding the role of gender on inflammation biomarker levels. 

The lack of distinct association between PM_2.5_ and inflammatory biomarkers, and adhesion molecules in this study does not exclude the possibility of chronic effects on the pulmonary inflammation and the cardiovascular system. Conclusions from the present study point to the complexity of explanatory variables, and limitations of sample size and short duration of study (i.e., 5–6 days per participant).

### 4.3. Limitations

This study has several limitations. Serum samples were stored in a freezer (−20 °C) at Kathmandu and were stored inside a freezer (−80 °C) only after arriving at the University of Massachusetts, Amherst. No quality checks were conducted to assess sample degradation due to storage at higher temperature. The study was conducted from the subjects who have unusually high levels of daily occupational exposure, and therefore likely reflects results specifically to individuals who have extreme exposures. The study was also complicated by not having the same individuals followed during both seasons. There are several variables that were untested in this study and these variables may be very important in affecting the health of the subjects. Perspiration, stress, and exhaustion might be high during hot summers than in cooler spring conditions. Psychological stress may also elevate inflammatory biomarkers such as IL-6 [59], though stress induced by busy traffic was not likely much different between spring and summer in this cohort as traffic patterns were generally unchanged. There was also an issue of compliance on wearing masks in summer because comfort levels in wearing masks were lower during summer than in spring. Respiratory allergies and illnesses which were not included in this study may also be playing an important role. Additionally, average 24-hour PM_2.5_ used for daily exposure may not be the right indicator. Alternatively, a higher percentile closer to the peak occupational exposure may more accurately reflect the highly polluted environments of day-time traffic. There can be various other factors that were not considered in the current regression model such as age, lifestyles and years on the job. Though the subjects were living together at the dormitory at the sampling site during the study and their residential exposures were considered in this study, the prior residential history may still have played a role in their health. For example, female traffic police might have been using biomass for cooking activities in the past, and such history was not noted and considered in the current study.

## 5. Conclusions

Though air pollution exposure levels were higher in spring than in summer, measured biomarkers levels were higher during summer than in spring. The results showed that cytokine expression (biomarker levels) did not show dependency only with personal PM_2.5_ exposure levels among traffic police and there may be other unmeasured factors such as genetics and stress. In general, short-term daily PM_2.5_ exposure levels solely affected half of measured biomarker concentrations in a highly polluted environment in the Kathmandu Valley. Particularly, PM_2.5_ had a mixed effect on these measured biomarker concentrations (positive association with 3 biomarkers and negative association with 4 biomarkers). Among the six variables tested for the statistical effect on individual biomarkers, season and gender were the most important variables affecting biomarker concentrations among traffic volunteers. This study has several limitations and further study is recommended to continue the investigation on potential factors that impact inflammation biomarkers.

## Figures and Tables

**Table 1:** Description of study subjects and air pollution during spring (*n* = 33) and summer (*n* = 29).

Parameter	Spring		Summer		
	Mean	SD	Mean	SD	*p*-value ^4^
a. Description of subjects ^1^					
BMI (kg/m^2^)	32	5	32	3	
Age	28	5	28	5	
Employment (years)	5	3			
b. Air pollution measurements					
PM_2.5_ (μg/m^3^) ^2^	123.51	38.66	45.21	24.09	0.000
BC (μgC/m^3^) ^2^	18.80	7.68	16.46	7.52	0.124
Passive sampling ^3^					
Ozone (μg/m^3^)	14.02	9.57	16.6	6.52	0.633
Sulfur dioxide (μg/m^3^)	6.75	0.35	25.46	10.99	0.098
Nitrogen dioxide (μg/m^3^)	103.94	15.65	102.02	51.21	0.939
Nitric oxide (μg/m^3^)	134.03	41.64	126.33	94.30	0.903

^1^ Based on subject samples in spring (*n* = 33) and summer (*n* = 29); ^2^ 24-hour average; ^3^ Passive sampling data are the one-week mean concentration from five sites [39]. ^4^ *p*-values are the results of two-sided independent *t*-tests.

**Table 2:** Comparisons of biomarker concentrations for traffic volunteers in Kathmandu between spring and summer, 2014.

Biomarkers	Independent Samples						Dependent Samples				
	Summer		Spring		Independent *t*-Test		Summer–Spring		*t*-Test		Wilcox Test
	Mean	Std. Dev.	Mean	Std. Dev.	*t* stat.	*p*-value	Mean Diff.	Std. Dev. Diff.	*t* stat	*p*-value	*p*-value
CRP ^1^	4.11	3.26	2.47	2.59	1.91	0.064	1.98	2.71	2.53	0.028 *	0.042 *
SAA ^1^	2.16	2.29	0.81	1.11	2.59	0.014 *	1.49	1.76	2.94	0.013 *	0.016 *
ICAM-1 ^1^	0.83	0.41	0.64	0.21	2.07	0.046 *	0.15	0.53	0.96	0.356	0.380
VCAM-1 ^1^	0.76	0.39	0.59	0.21	1.77	0.086	0.18	0.51	1.22	0.249	0.176
IFN-γ ^2^	25.53	7.89	17.76	7.73	2.56	0.014 *	5.58	8.89	2.17	0.052	0.064
IL-1β ^2^	3.03	1.94	1.46	0.28	3.91	0.001 ***	0.59	0.53	3.85	0.003 **	0.001 ***
IL-2 ^2^	7.42	2.41	3.53	1.35	6.89	0.000 ***	4.96	3.16	5.43	0.000 ***	0.001 ***
IL-4 ^2^	0.37	0.11	0.19	0.08	6.34	0.000 ***	0.18	0.09	6.84	0.000 ***	0.001 ***
IL-6 ^2^	2.26	0.82	0.89	0.40	7.33	0.000 ***	1.21	0.71	5.93	0.004 **	0.001 ***
IL-8 ^2^	64.29	63.54	12.98	4.76	3.78	0.001 **	78.93	74.56	3.67	0.001 ***	0.001 ***
IL-10 ^2^	2.31	0.59	1.28	0.62	5.83	0.000 ***	1.13	0.81	4.85	0.008 **	0.002 **
IL-12 ^2^	2.18	0.51	1.42	0.43	5.56	0.000 ***	0.61	0.65	3.25	0.003 **	0.012 *
IL-13 ^2^	1.49	0.59	0.56	0.34	6.64	0.000 ***	0.62	0.57	3.78	0.008 **	0.005 **
TNF-α ^2^	5.16	2.09	3.23	1.19	3.90	0.000 ***	1.79	2.15	2.88	0.015 *	0.001 **

Concentrations: ^1^ µg/mL; ^2^ pg/mL; level of significance: * *p* < 0.05; ** *p* < 0.01; *** *p* < 0.001. All the tests were two-sided and conducted on the data of each individual’s average biomarker concentrations. The independent *t*-test performed a two-sample *t*-test, assuming independence of the volunteer samples in the two seasons. Dependent samples only counted the subjects with biomarker measurements in both seasons. Both a parametric *t*-test and a nonparametric Wilcox test were performed on the dependent samples.

**Table 3:** Effects estimate from linear mixed models.

Effects	Categories	Estimate	Std. Error	DF	*t* Value	Pr > |*t*|
CRP ^1^						
Season	Summer	0.3462	0.0616	53	1.90	0.0630
PM_2.5_		0.0010	0.0005	53	5.62	<0.0001
SAA ^2^						
Season	Summer	1.4828	0.2742	59	5.41	<0.0001
PM_2.5_		0.0029	0.0026	59	1.13	0.2637
Gender	Female	0.9548	0.3167	59	3.01	0.0038
VCAM-1 ^2^						
Season	Summer	0.3154	0.0908	59	3.47	0.0010
ICAM-1 ^2^						
Season	Summer	0.3679	0.0831	70	4.43	<0.0001
IL-1β ^2^						
Season	Summer	0.3829	0.1124	67	3.41	0.0011
PM_2.5_		−0.0018	0.0007	67	−2.45	0.0171
IL-2 ^2^						
Season	Summer	0.8941	0.06547	72	13.59	<0.0001
Gender	Female	0.5940	0.1334	72	4.45	<0.0001
IL-4						
Season	Summer	0.1812	0.0154	66	11.77	<0.0001
Gender	Female	0.2077	0.01931	66	10.76	<0.0001
IL-6 ^2^						
Season	Summer	0.9458	0.08211	71	11.52	<0.0001
Gender	Female	0.6877	0.1463	71	4.70	<0.0001
IL-8 ^2^						
Season	Summer	1.1651	0.1411	59	8.26	<0.0001
IL-10						
Season	Summer	1.2331	0.1417	57	8.71	<0.0001
PM_2.5_		0.0028	0.0009	57	3.07	0.0033
BC		−2 × 10^−5^	4.44 × 10^−6^	57	−3.55	0.0008
Mask	No	−0.1199	0.0477	57	−2.52	0.0147
Gender	Female	1.1191	0.2337	57	4.79	<0.0001
IL-12						
Season	Summer	0.6483	0.1048	62	6.19	<0.0001
PM_2.5_		−0.0021	8.56 × 10^−4^	62	−2.42	0.0184
Mask	No	−0.2006	0.0506	62	−3.97	0.0002
Gender	Female	0.8294	0.1720	62	4.82	<0.0001
IL-13						
Season	Summer	0.6104	0.1014	58	6.02	<0.0001
PM_2.5_		−0.0019	0.0005	58	−4.08	<0.0001
Mask	No	−0.0963	0.0297	58	−3.24	0.0020
Gender	Female	0.9112	0.0576	58	15.81	<0.0001
IFN-γ ^3^						
PM_2.5_		−0.0044	0.0014	68	−3.22	0.0019
Gender	Female	0.9682	0.2299	68	4.21	<0.0001
TNF-α						
Season	Summer	1.4738	0.2158	65	6.83	<0.0001
BC		−5.00 × 10^−5^	1.60 × 10^−5^	65	−3.23	0.0020
Smoker	No	−0.7800	0.3269	65	−2.39	0.0200

^1^ The 4th root of the variable was used in the model. ^2^ The log of the variable was used in the model. ^3^ The square root of the variable was used in the model.

## Supplementary Materials

The following are available online at http://www.mdpi.com/1660-4601/16/3/377/s1. The strategy diagrams for building linear mixed models and variation of air pollution among six sites are given in supplementary information. Figure S1. Strategy diagram for building linear mixed models. Table S1. Variation of personal air pollution exposure at six sites between two seasons.
