# Supplementary material for: Roadside Exposure and Inflammation Biomarkers among a Cohort of Traffic Police in Kathmandu, Nepal
Source: Int J Environ Res Public Health. 2019 Jan 29;16(3):377. doi: 10.3390/ijerph16030377 (PMC6388290; doi:10.3390/ijerph16030377)
Supplement: Supplementary file 1 [file ijerph-16-00377-s001.pdf]

Supplementary Information

Roadside exposure and inflammation biomarkers among a cohort of traffic police in Kathmandu, Nepal

Kabindra M. Shakya[1], Richard E. Peltier[2], Yimin Zhang[3], Basu D. Pandey[4]

[1]Department of Geography and the Environment, Villanova University, Pennsylvania

[2]Department of Environmental Health Science, University of Massachusetts, Amherst

[3]Department of Mathematics and Statistics, Villanova University, Pennsylvania

[4]Sukraraj Tropical and Infectious Disease Hospital, Kathmandu and Everest International Clinic and Research Center, Nepal

[1]Corresponding author

800 Lancaster Avenue, Villanova, PA 19085, USA

Email: kabindra.shakya@villanova.edu

Phone: 610-519-3590

Fax: 610-519-3338

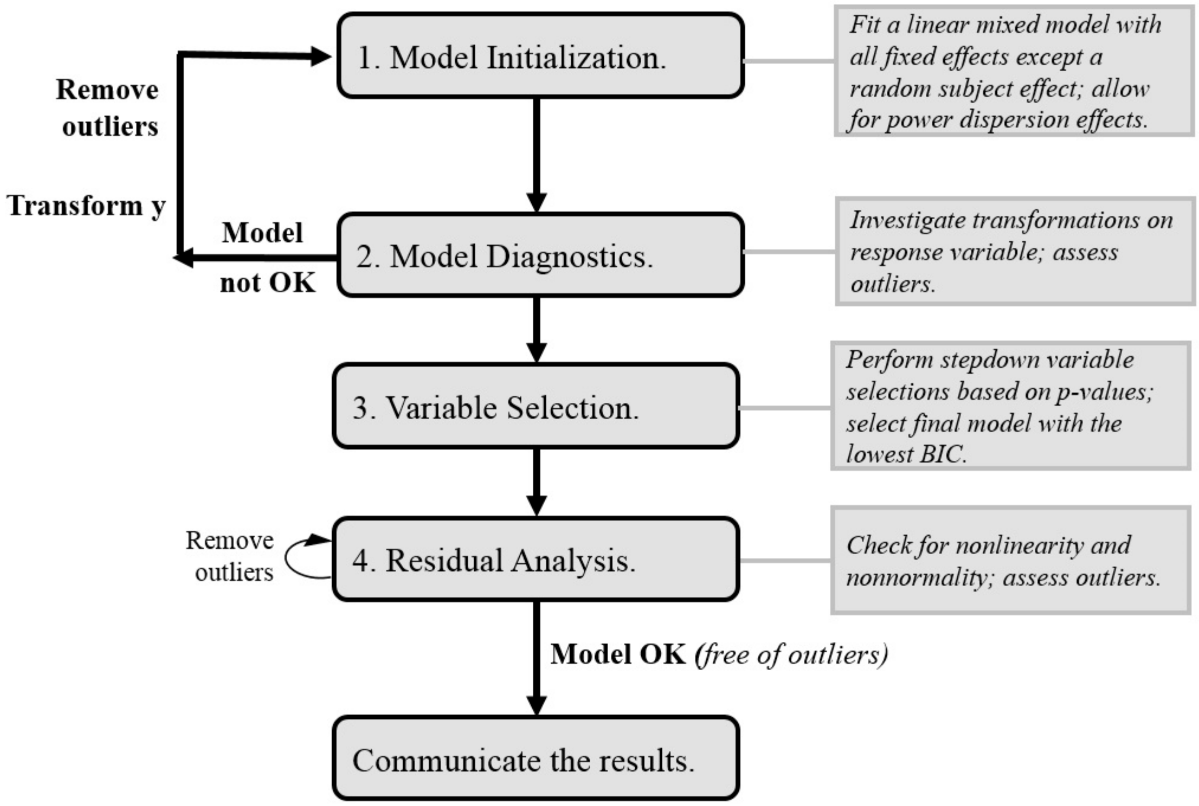

Figure S1. Strategy diagram for building linear mixed models.

Table S1. Variation of personal air pollution exposure at six sites between two seasons.

**Site**	**$PM_{2.5}$**		**BC**	
	Spring	Summer	Spring	Summer
Balaju	144.44 ± 29.94	65.69 ± 22.55	22.61 ± 4.76	20.19 ± 7.63
Chabahil	130.82 ± 23.73	48.92 ± 21.84	23.90 ± 9.05	14.71 ± 7.47
Jawalakhel	92.22 ± 44.98	44.43 ± 23.21	14.10 ± 2.28	15.72 ± 7.03
Kalanki	168.21 ± 28.41	-	18.60 ± 3.23	-
Thapathali	108.39 ± 28.79	24.33 ± 63.38	11.97 ± 5.88	12.37 ± 5.70

Mean and one standard deviation of 24-hour average of exposure for the personnel who volunteered to provide blood samples.
